# Data on molecular characterisation and expression analysis of the interferon-related developmental regulator 2 (IFRD2) gene from red sea bream, *Pagrus major*

**DOI:** 10.1016/j.dib.2019.104142

**Published:** 2019-06-12

**Authors:** Kyung Ho Kim, Andre Kim, Chan-Il Park

**Affiliations:** a Institute of Marine Industry, College of Marine Science, Gyeongsang National University, 455, Tongyeong, 650-160, Republic of Korea; b Department of Pharmaceutical Engineering, College of Medical and Life Science, Silla University, Busan, 617-736, South Korea

**Keywords:** Interferon-related developmental regulator 2 (IFRD2), Red sea bream, *Edwardsiella piscicida*, *Streptococcus iniae*, Red sea bream iridovirus (RSIV)

## Abstract

The interferon-related developmental regulator 1 (IFRD1) protein is expected to play a role in the regulation of inflammatory responses in adult mice, since it is known to repress transcription of NF-κB in myoblasts that regenerate skeletal muscle after traumatic injury Micheli et al., 2011. The IFRD2 gene is expressed in many tissues including skeletal muscle, kidney, heart, brain, lung, placenta and liver in adult humans and is highly expressed in adult human skeletal muscle and heart. In mice, interferon-related developmental regulator 2 (IFRD2) may be associated with early haematopoiesis after gastrulation and in the hepatic primordium Buanne et al., 1998. In this study, we analysed the molecular characteristics of the IFRD2 gene identified from *Pagrus major* (PmIFRD2) and performed multiple alignments and phylogenetic analyses of the protein sequence. In addition, we examined the expression pattern of IFRD2 in healthy red sea bream tissues and the temporal expression pattern after challenging with various pathogens [*Edwardsiella piscicida* (*E. piscicida*), *Streptococcus iniae* (*S. iniae*) and red sea bream iridovirus (RSIV)]. This study characterises the non-specific immune response of the red sea bream after viral and microbial infections.

**Table:** Specifications Table

Subject area	*Immunology and Microbiology*
More specific subject area	*Gene expression analysis*
Type of data	*Figure*
How data was acquired	*Expression analysis was performed by real-time polymerase chain reaction [(Thermal Cycler Dice Real-Time System (TaKaRa Bio Inc)]. Multiple sequence alignment was performed using the program Basic Local Alignment Search Tool (BLASTX) from the National Center for Biotechnology Information (NCBI) and the GENETYX ver. 7.0. program. Phylogenetic classification was performed using the program Mega 4.0. Positions of signal peptides and protein domain were confirmed using the Simple Modular Architecture Research Tool (SMART) and signalP program. The molecular weight (MW) and isoelectric point (pI) were predicted using the ProtParam tool from the ExPASy Proteomics server.*
Data format	*Analysed and Real-time PCR*
Experimental factors	*Open reading frame (ORF) of PmIFRD2 cDNA was obtained from next generation sequencing (NGS) analysis from liver of rea sea bream challenged with S. iniae. PmIFRD2 gene expression level profiles were compared between healthy fish and fish challenged with various pathogens.*
Experimental features	*This experiment could be provided as a basis for analysing the functional characteristics of the PmIFRD2 gene in the non-specific immune system of red sea bream.*
Data source location	*Gyeongsang National University, Tongyeong, Republic of Korea*
Data accessibility	*The data are available for this article*

**Table:** 

**Value of the data** • These data provide a basis for predicting the function of IFRD2 through phylogenetic analysis of IFRD2 in *Pagrus major* and other species. • These data provide a basis for understanding the role of PmIFRD2 in the immune system of red sea bream infected with various pathogens. • PmIFRD2 mRNA expression analysis results can also be used in comparative analyses of IFRD2 gene expression in other fish species.

## 1 Data

**Fig. 1:**
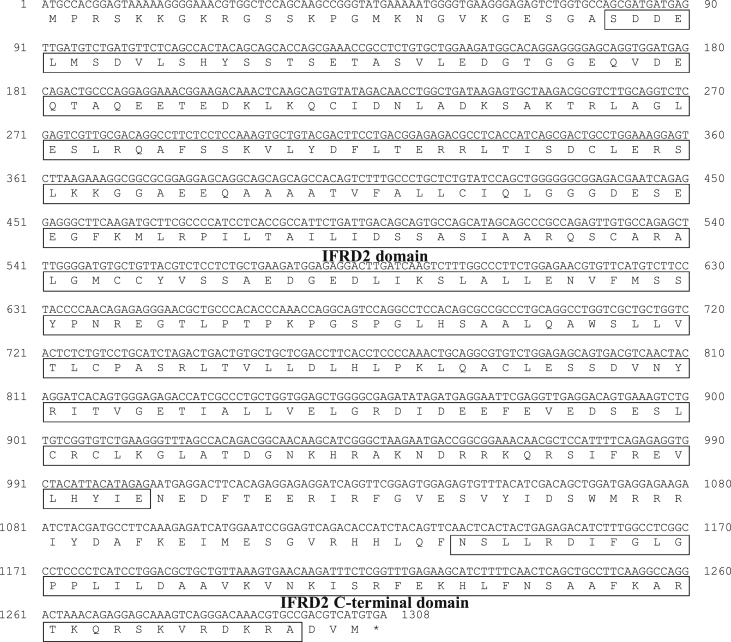
cDNA and deduced amino acid sequence of PmIFRD2. The IFRD2 domain and IFRD2 C-terminal domain are indicated by a box.

**Fig. 2:**
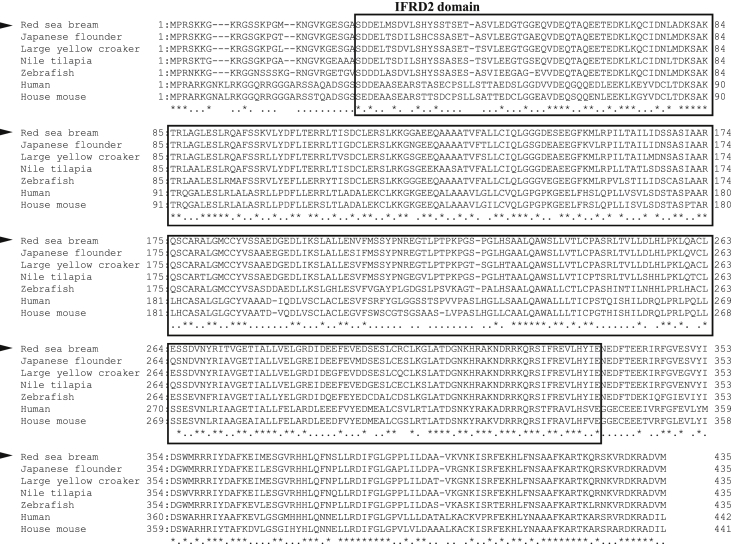
Multiple alignments comparing PmIFRD2 with IFRD2 amino acid sequences from other species. The NCBI accession numbers of dicentracin are as follows: Large yellow croaker (XP_010740526); Japanese flounder (XP_019944902); Nile tilapia (XP_003444802); Zebrafish (NP_001003621); House mouse (NP_080179); Human (AAV38802).

**Fig. 3:**
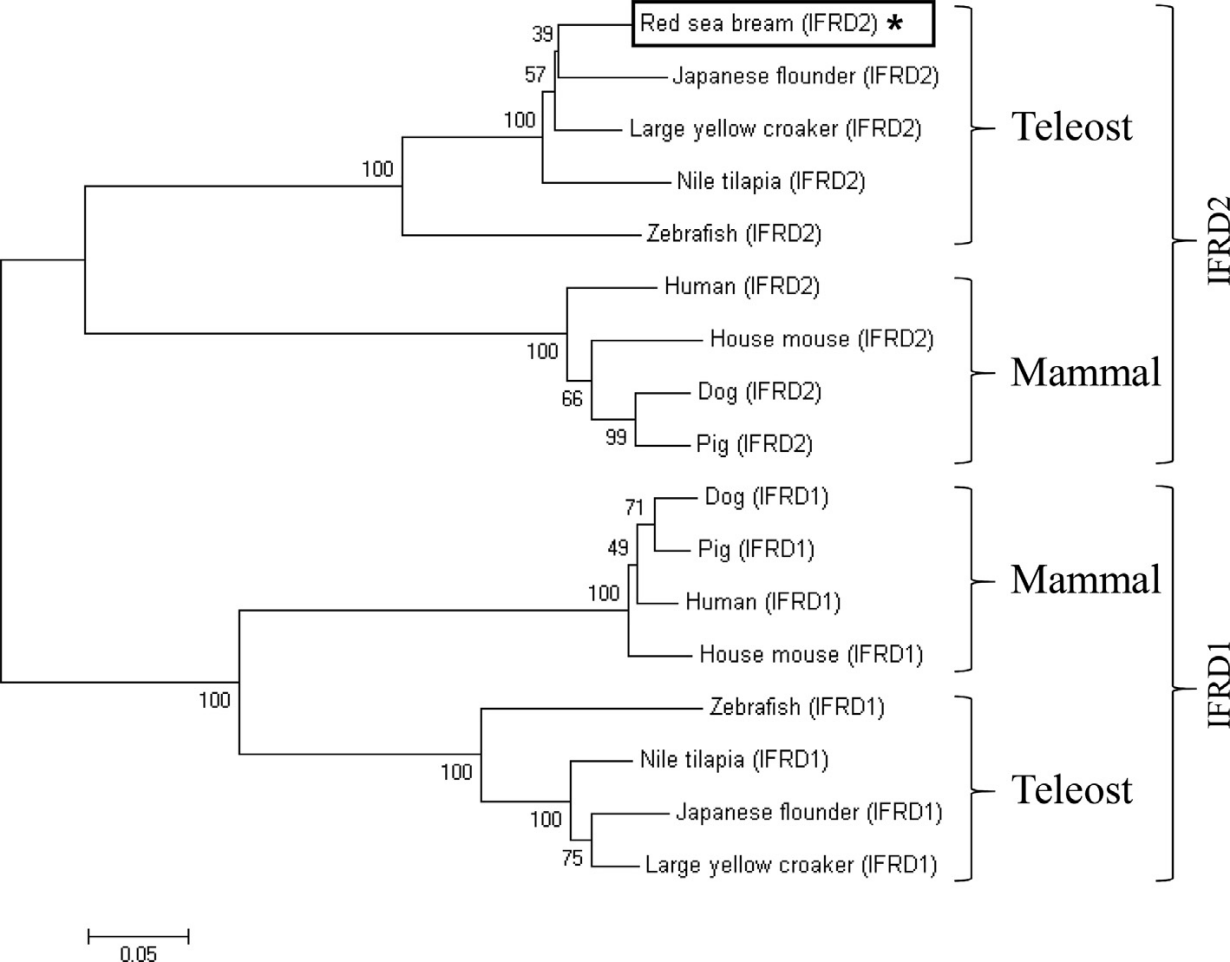
Phylogenetic analysis of deduced IFRD2 amino acid sequences in other species. The phylogenetic tree was constructed using the neighbour-joining method in MEGA 4 software. Bootstrap sampling was performed with 2000 replicates. The scale bar is equal to 0.05 changes per amino acid position.

**Fig. 4:**
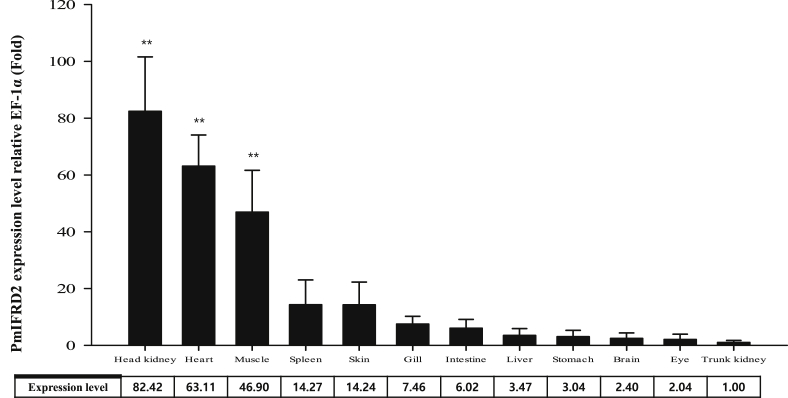
Detection of PmIFRD2 mRNA expression in various tissues from healthy red sea bream by real-time PCR. EF-1α was used to normalise the real-time PCR results. Data are presented as the mean ± SD from three independent cDNA samples with three replicates from each sample. Asterisks indicate significant differences (**P value < 0.01) compared to the trunk kidney.

**Fig. 5:**
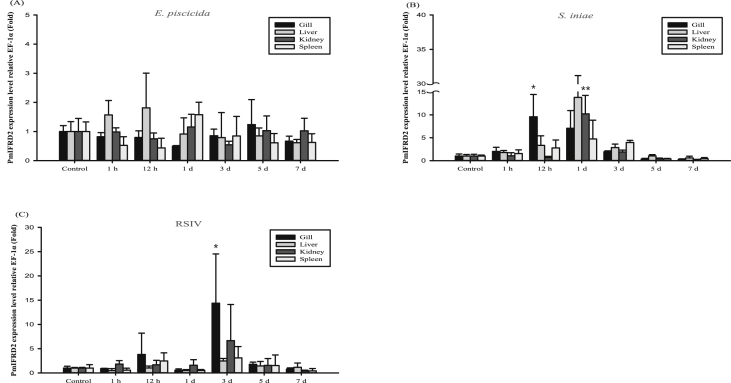
PmIFRD2 mRNA expression levels in various tissues of red sea bream infected with three pathogens: (A) *Edwardsiella piscicida* (*E. piscicida*), (B) *Streptococcus iniae* (*S. iniae*) and (C) red sea bream iridovirus (RSIV). The levels of PmIFRD2 transcripts were normalised to EF-1α levels. The data are presented as the mean ± SD from three independent cDNA samples with three replicates for each sample. The asterisks represent significant differences compared to the control (PBS) group by ANOVA (*P value < 0.05 and **P value < 0.01).

The interferon-related developmental regulator 1 (IFRD1) protein has been reported to play a role in the regulation of inflammatory responses [1]. Also, interferon-related developmental regulator 2 (IFRD2) in mice may be associated with early haematopoiesis after gastrulation and in the hepatic primordium [2]. The open reading frame (ORF) of the PmIFRD2 cDNA was identified from red sea bream injected with *S. iniae* and consisted of 1308 bp, that encoded 435 amino acids (aa). The predicted domains of IFRD2 included the IFRD2 domain (27–335 aa) and the IFRD2 C-terminal domain (380–432 aa) (Fig. 1). The isoelectric point and molecular weight of the PmIFRD2 protein were predicted to be 6.0 and 48.2 kDa, respectively. Multiple alignment analyses of the IFRD2 amino acid sequences between red sea bream, and other teleosts and mammals revealed that IFRD2 from the large yellow croaker was the most homologous to PmIFRD2 at 92.64%. Among the teleosts, IFRD2 from the zebrafish was the least homologous to PmIFRD2 (78.49%), and IFRD2 from house mouse (53.85%) was the least homologous to PmIFRD2 among mammals (Fig. 2). To confirm the phylogenetic location of PmIFRD2, the phylogenetic tree was divided into teleost and mammalian clusters, and PmIFRD2 was most closely related to the Japanese flounder and large yellow croaker in the teleost cluster (Fig. 3). Quantitative real-time PCR (RT-qPCR) was used to confirm the expression levels of PmIFRD2 mRNA in healthy and infected red sea breams. The expression analysis of PmIFRD2 mRNA in healthy red sea bream, showed 82.42-fold more expression in the head kidney than in the trunk kidney (Fig. 4). The expression patterns of PmIFRD2 mRNA in gills, liver, kidney and spleen were confirmed after challenging red sea breams with *E. piscicida*, *S. iniae* or RSIV (Fig. 5). After challenging with *E. piscicida*, the expression of PmIFRD2 mRNA was slightly elevated in the liver at 1 hour post-infection (hpi) and increased to 1.81-fold at 12 hpi. In the spleen, the expression was 1.57-fold higher at 1 day post-infection (dpi), but there was no significant difference (Fig. 5-A). After infection with *S. iniae,* the expression of PmIFRD2 was significantly upregulated in the gills at 12 hpi, in the kidney at 1 dpi, and in the liver at 12 hpi and 7 dpi (Fig. 5-B). After challenging with RSIV, the expression of PmIFRD2 mRNA was significantly upregulated in the gills at 3 dpi (Fig. 5-C).

## 2 Experimental design, materials, and methods

### 2.1 Sequence and phylogenetic analysis of PmIFRD2

PmIFRD2 was acquired from the liver of a red sea bream and the ORF identified by NGS analysis. Sanger sequencing was performed to verify the cDNA sequence of PmIFRD2. The amino acid sequence of PmIFRD2 was predicted using the GENETYX ver. 7.0 program (SDC Software Development, Japan) and the NCBI BLAST program. The molecular weight and isoelectric point of PmIFRD2 were predicted using the ProtParam tool of the ExPASy Proteomics Server, and the location of the specific domains of PmIFRD2 was predicted using SMART web software. Multiple sequence alignments were analysed by ClustalW between the predicted amino acid sequence of PmIFRD2 and the IFRD2 amino acid sequences of other species registered in the NCBI peptide sequence database. In addition, the phylogenetic analysis of PmIFRD2 was performed using the neighbour-joining (NJ) method of the Mega 4 program. Support for each node was derived from 2000 bootstrap replicates.

### 2.2 RT-qPCR analysis of PmIFRD2

#### 2.2.1 Fish

Experimental healthy red sea bream (weight: 68.5 ± 10 g, body length: 14.3 ± 1 cm) were supplied by Gyeongsangnam-do Fisheries Resources Research Institute (Tongyeong, Republic of Korea), kept in a seawater tank (water temperature: 20–23 °C) for 2 weeks and fed daily on a commercial diet during the acclimatisation period. Three healthy red sea bream were anaesthetised with benzocaine (Sigma, USA) before tissue collection. For the bacterial and viral challenge experiment, *S. iniae* (1.5 × 10^5^ CFU/fish), *E. piscicida* (1.5 × 10^5^ CFU/fish) or RSIV (1 × 10^5^ copies/fish) were obtained from the Fish Pathology Division of the National Institute of Fisheries Science (Busan, Republic of Korea).

#### 2.2.2 Various tissue samples of healthy fish for PmIFRD2 mRNA expression analysis

Three healthy red sea bream were anaesthetised, and 12 tissues were sampled including the head kidney, heart, muscle, spleen, skin, gills, intestine, liver, stomach, brain, eye and trunk kidney, which were aseptically isolated to profile tissue mRNA expression. All samples were stored immediately frozen in liquid nitrogen at −80 °C until they were used for total RNA extraction.

#### 2.2.3 Total RNA extraction and cDNA synthesis

Total RNA was extracted from various red sea bream tissues using TRIzol reagent (Invitrogen, USA) according to the manufacturer's instructions. Briefly, 500 μL of TRIzol was added to each sample and then homogenised. A total of 100 μL of chloroform (Invitrogen) was added, and the samples were vortexed and centrifuged at 14,000 rpm for 10 min. The supernatant was transferred to a new 1.5-mL tube, equilibrated with PCI (phenol:chloroform:isoamyl alcohol) and centrifuged at 14,000 rpm for 10 min. The supernatant was transferred to a new 1.5-mL tube and then mixed with 500 μL of isopropanol (Sigma), 5 μL of Dr. Gen (TaKaRa, Japan), and 30 μL of 3 M sodium acetate (TaKaRa) and then centrifuged at 14,000 rpm for 10 min. After removing the supernatant, 600 μL of 75% DEPC ethyl alcohol was added and centrifuged at 14,000 rpm for 5 min. Finally, the supernatant was removed, the RNA was allowed to dry naturally at room temperature for 10–15 min, and then it was resuspended in 30–40 μL of DEPC DDW. After extraction of total RNA, samples were treated with RNase-free DNase (Promega, USA) according to the manufacturer's instructions. cDNA synthesis was carried out using the PrimeScript™ 1st strand cDNA Synthesis Kit (Takara) according to the manufacturer's instructions.

#### 2.2.4 RT-qPCR analysis

The tissue expression profile of PmIFRD2 mRNA was assayed by RT-qPCR with a DICE Real-Time System Thermal Cycler (TaKaRa). The specific primer sets were designed by Primer3 ver. 3 (http://bioinfo.ut.ee/primer3-0.4.0/) based on the cDNA sequence of PmIFRD2 (forward: 5′-CATCCTCACCGCCATTCT-3′, reverse: 5′-AGTCCTCTCCATCTTCAGCA-3′). For RT-qPCR, 1 μL of cDNA template, 1 μL of forward and reverse primers, 9.5 μL of DDW and 12.5 μL of TB Green were mixed in a total volume of 25 μL using TB Green premix Ex Taq™ (TaKaRa). The cDNA mixture was used in the following reaction, conditions: incubation for 4 min at 50 °C, an initial denaturation step for 10 min at 95 °C, and then 45 cycles of 20 s at 95 °C and 30 s at 60 °C, followed by a final dissociation stage for 15 s at 95 °C, 30 s at 60 °C and 15 s at 95 °C. The degree of PmIFRD2 mRNA expression was compared with the expression level of elongation factor 1 alpha (EF-1α) (forward: 5′-CCTTCAAGTACGCCTGGGTG-3′, reverse: 5′-CTGTGTCCAGGGGCATCAAT-3′) mRNA and three repetitions were performed for each gene for the accuracy of the experiment. The relative mRNA expression levels were calculated using the comparative Ct (2^−ΔΔCT^) method and normalised to EF-1α.

#### 2.2.5 Expression of PmIFRD2 after challenge with pathogens

Healthy red sea bream were randomly divided into three groups and then challenged by intraperitoneal injection with 100 μL of *S. iniae* (1.5 × 10^5^ CFU/fish), *E. piscicida* (1.5 × 10^5^ CFU/fish) or RSIV (1 × 10^5^ copies/fish) suspension, respectively. The control group was injected with the same volume of phosphate buffered saline (PBS). The gills, liver, kidney and spleen from the three fish at 1 and 12 hpi, and 1, 3, 5 and 7 dpi from each group were isolated and frozen in liquid nitrogen. All samples were obtained and analysed in triplicate, and total RNA extraction, cDNA synthesis and RT-qPCR were performed as described above.
